# Novel Ion Channel Genes in Malaria Parasites

**DOI:** 10.3390/genes15030296

**Published:** 2024-02-26

**Authors:** Sanjay A. Desai

**Affiliations:** Laboratory of Malaria and Vector Research, National Institute of Allergy and Infectious Diseases, National Institutes of Health, Rockville, MD 20852, USA; sdesai@nih.gov

**Keywords:** ion channels, nutrient uptake, protein export, transmembrane transport, malaria, *Plasmodium falciparum*, antimalarial therapies

## Abstract

Ion channels serve many cellular functions including ion homeostasis, volume regulation, signaling, nutrient acquisition, and developmental progression. Although the complex life cycles of malaria parasites necessitate ion and solute flux across membranes, the whole-genome sequencing of the human pathogen *Plasmodium falciparum* revealed remarkably few orthologs of known ion channel genes. Contrasting with this, biochemical studies have implicated the channel-mediated flux of ions and nutritive solutes across several membranes in infected erythrocytes. Here, I review advances in the cellular and molecular biology of ion channels in malaria parasites. These studies have implicated novel parasite genes in the formation of at least two ion channels, with additional ion channels likely present in various membranes and parasite stages. Computational approaches that rely on homology to known channel genes from higher organisms will not be very helpful in identifying the molecular determinants of these activities. Given their unusual properties, novel molecular and structural features, and essential roles in pathogen survival and development, parasite channels should be promising targets for therapy development.

## 1. Introduction

Malaria parasites are successful single-cell eukaryotic pathogens of humans, other primates, rodents, birds, and reptiles. In humans, five *Plasmodium* species cause malaria and are responsible for significant morbidity and mortality; they also continue to exact a staggering toll on the global economy through reduced productivity and compromised childhood development.

**Figure 1:**
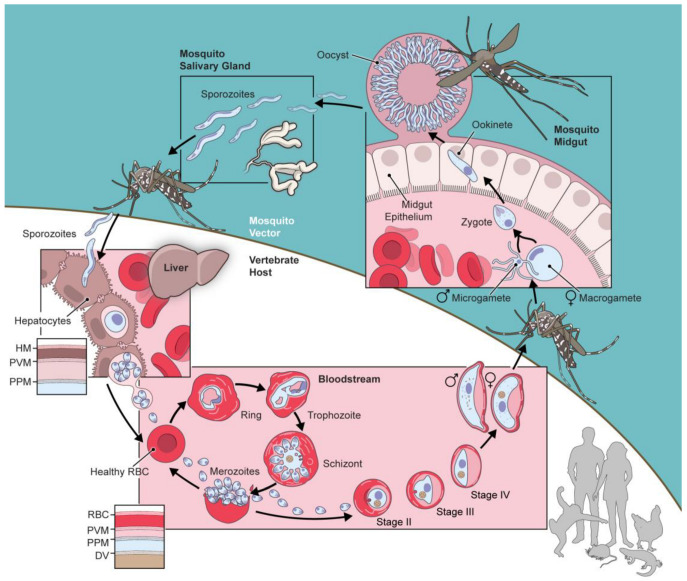
Schematic showing the life cycle of *Plasmodium* spp. in a vertebrate host and mosquito vector. While some stages shown are specific to *P. falciparum*, genus members infecting primates, rodents, birds, and lizards have similar life cycles with multiple intracellular and extracellular forms in both the host and the vector. During intracellular development, there are multiple membranous barriers to ion and nutrient exchange, as highlighted with insets for infected erythrocytes and hepatocytes. HM, hepatocyte plasma membrane; PVM, parasitophorous vacuolar membrane; PPM, parasite plasma membrane; RBC, red blood cell; DV, digestive vacuole.

The success of the most virulent human pathogen, *P. falciparum*, results in part from its complicated life cycle, which allows for cycles of exponential replication in multiple stages (Figure 1). A key feature of this life cycle is the presence of extracellular and intracellular parasite forms in both the vertebrate host and the mosquito vector. In the host bloodstream, for example, the parasite invades and replicates within circulating erythrocytes. This intracellular habitat provides access to erythrocyte hemoglobin as an amino acid source; it also facilitates immune evasion as the sequestered parasite is hidden from immune cells and soluble effectors.

Another important feature apparent in the parasite life cycle is the presence of multiple membrane barriers to ion and solute exchange with the extracellular environment. As with other eukaryotic cells, parasite growth and replication depend on this solute exchange. The uptake of extracellular nutrients such as amino acids, sugars, and precursors of nucleic acid and phospholipid biosynthesis is essential [1,2,3,4,5,6], especially in bloodstream forms as key nutrients are not adequately present in the erythrocyte cytosol to fuel rapid parasite growth and replication (Figure 1). In addition to nutrient uptake, ions must also be transported across these various membranes. Ca^++^ uptake, for instance, is required for developmental progression and DNA replication in asexual parasite forms [7,8,9,10]; this divalent cation must be acquired in the face of an efficient plasma membrane Ca^++^ ATPase (PMCA) extrusion pump that maintains a remarkably low intracellular [Ca^++^] in erythrocytes [11,12]. The transmembrane flux of other ions such as Na^+^, K^+^, and Cl^−^ is also essential for cell volume regulation, ion homeostasis, the establishment of membrane potentials, and signaling [13]. These needs are further exacerbated for intracellular parasites because ions and other solutes must cross multiple membranes to permit survival and growth. 

Because the large-scale transport of ions and organic solutes is often mediated by ion channels, it was surprising and unexpected that the completion of *P. falciparum* genome sequencing revealed very few ion channel genes (Table 1). These genes were identified through homology to known channel genes from higher organisms [14]. Notably absent are conventional Ca^++^ channels despite the importance of Ca^++^ uptake in parasite growth and development; the absence of Cl^−^ channels was also striking during genome assembly. 

This review focuses on parasite ion channels and their genes; Table 1 does not include transporters and pumps, which are not considered further in this review. Channels are distinguished from transporters in that they allow for higher rates of transport (>10^10^ ions/second) [15]. Channel-mediated flux is passive because ions and/or solutes move down their electrochemical gradients through a water-filled pore. While transporters can serve similar biological roles, their lower rates of transport make biochemical studies, such as with the patch-clamp technique [16,17], more difficult. 

**Table 1:** *P. falciparum* ion channel genes identified by homology to channel genes in other organisms. For each gene, the number of predicted transmembrane domains (#TMD) was determined using DeepTMHMM [18].

Gene	Annotation	#TMD	References
PF3D7_1227200	potassium channel K1	8	[19,20,21]
PF3D7_1465500	potassium channel K2	8	[20,22]
PF3D7_1107900	small-conductance mechanosensitive ion channel (MscS)	6	[23]
PF3D7_1432100	VDAC	0	[24]
PF3D7_1250200	CSC1-like protein, putative	11	[25]
PF3D7_0810400	aquaporin	2	[26]
PF3D7_1132800	aquaglyceroporin	6	[27,28,29]
PF3D7_0408700	perforin-like protein 1	0	[30,31,32]
PF3D7_1216700	perforin-like protein 2	0	[30,33]
PF3D7_0923300	perforin-like protein 3	0	[34]
PF3D7_1473700	nucleoporin NUP116/NSP116, putative	0	[35]

Despite the paucity of ion channel genes uncovered via the whole-genome sequencing of *Plasmodium* spp., patch-clamp and other transport assays have identified several unusual ion channels in blood-stage malaria parasites. Here, I review the discovery and properties of these channels and discuss insights into their molecular basis. These insights reveal that malaria parasites have unique channels encoded by genes absent from higher organisms. These unusual microbial channels serve essential roles in parasite biology and development and are, therefore, important targets for antimalarial therapies. Their study can also provide foundational insights into solute recognition and permeation.

## 2. The Plasmodial Surface Anion Channel (PSAC)

### 2.1. Background

**Figure 2:**
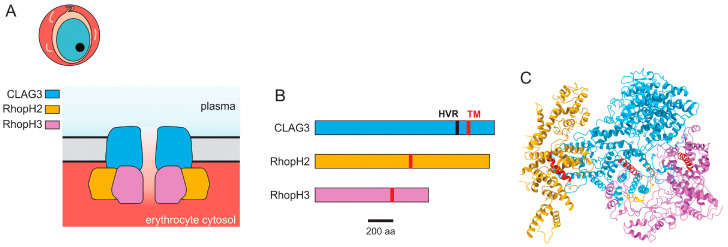
The plasmodial surface anion channel (PSAC). (**A**). Model for PSAC formation by subunits of the RhopH complex. CLAG3 is exposed at the erythrocyte surface, while RhopH2 and RhopH3 are endofacial proteins. (**B**). Ribbon diagrams of RhopH proteins, drawn to scale. The positions of the single transmembrane domain predicted for each subunit are indicated in red; CLAG3 has a hypervariable region (HVR) exposed at the host cell surface. (**C**). Cryo-EM reconstruction of the soluble RhopH complex prior to insertion in the host membrane. Subunit color scheme as in panel (**B**), with α-helical transmembrane domains shown in red. PDB: 7KIY.

The plasmodial surface anion channel (PSAC) is a prime example of an unusual ion channel present only in *Plasmodium* spp. This channel is on the host erythrocyte membrane and serves an essential role in nutrient acquisition for the intracellular parasite (Figure 2A). It accounts for the increased permeability of infected erythrocytes to a broad range of organic and inorganic solutes [36], as first identified some 75 years ago and characterized using tracer accumulation, osmotic fragility, and other transport assays in numerous studies before 2000 [2,3,4,5]. 

These early studies had three key limitations, all of which arose because the transport methodologies depended on macroscopic flux measurements of populations of cells. First, the precise mechanism of solute uptake was unclear, with proposals including one or more parasite- or host-derived ion channels or transporters, lipid defects resulting from parasite invasion, fluid-phase endocytosis, and membranous ducts that could provide direct access to plasma [4,37,38,39]. Second, because endocytosis and membranous ducts were possible, macroscopic measurements could not determine the subcellular location of the solute flux across membranes. Finally, although there was substantial interest in identifying the molecular basis [40], these uncertainties prevented systematic studies aimed at gene identification. Because the location, mechanism, and required number of distinct routes were unclear, the original studies used “new permeability pathways” (NPPs) to describe infection-associated permeability increases [41].

The first advance in addressing these questions came from the introduction of patch-clamp methods. The first patch-clamp recordings of infected human erythrocytes identified the PSAC as an ion channel mechanism of transport [42]. Cell-attached patch-clamp experiments revealed individual channel molecules on infected erythrocytes that were conserved in divergent *Plasmodium* spp. [42,43]. Because this method could limit the measurement of ion flux to a small “patch” of the erythrocyte plasma membrane [44], it also established the host cell membrane as the location of the transport activity. This study also reported results using the whole-cell patch clamp method, allowing for a quantitative estimate of 1000–2000 functional channel molecules on a mature trophozoite- or schizont-infected cell. When combined with noise analyses of single-channel and whole-cell recordings [45], the study also determined that the PSAC was the predominant conductive pathway for ion flux on infected erythrocytes.

Interestingly, subsequent studies from other groups confirmed increased anion channel-mediated currents at the host membrane but suggested multiple distinct channel types [46,47,48,49]. These studies also proposed various regulators of channel activity, including activation by oxidative stress [48], cyclic nucleotides [47], membrane stretching [50], and a link to various mammalian ion channels [49,51].

The completion of *Plasmodium falciparum* whole-genome sequencing failed to identify orthologs of known anion channels [14], raising questions about the molecular basis of the identified channel(s). Because the synthesis, trafficking, and insertion of parasite proteins at the host membrane were also considered overly complicated, nearly all workers assumed that the observed channels were host proteins that become activated or modified by the intracellular parasite [46,52]. There was already evidence for the parasite modification of some erythrocyte membrane proteins [40,53,54], so this model appeared to be the most conservative one. 

### 2.2. Identification of the Rhoph Genes as PSAC Determinants

The unusual properties of the channel, nevertheless, prompted searches for parasite genetic elements involved in PSAC formation. The first experimental evidence supporting parasite genes was the identification of differences in the channel’s voltage-dependent gating [55], a term that encompasses the process of the opening and closing of the pore to ion flux. More compelling evidence included the identification of distinct PSAC mutants with altered solute flux, selectivity, single channel gating, and pharmacology [56,57,58]; these mutants were identified through in vitro selection with blasticidin S and/or leupeptin, antiparasitic toxins that require PSAC-mediated uptake to reach their intracellular targets. These studies suggested that continuous cultivation with these toxins selected for an outgrowth of mutants with reduced toxin uptake at the erythrocyte membrane.

Based on these findings, a library of >50,000 small molecules was screened for PSAC inhibitors that produce differential blocks of channels associated with geographically divergent parasite clones. High-throughput screens of four divergent clones and pairwise comparisons identified ISPA-28, a unique inhibitor that blocks channels associated with the Dd2 clone with a 800-fold higher affinity than those linked to the HB3 clone [36]. An available Dd2 × HB3 genetic cross was then used to track inheritance in 34 progeny clones, revealing that most daughter parasites produced channels matching one or the other parental line and providing conclusive evidence for parasite genetic elements. A linkage analysis implicated a single locus near the 5′ end of parasite chromosome 3. Because none of the genes in this locus resembled known ion channel genes, the Dd2 line was then transfected to produce merodiploid parasites expressing both the HB3 and Dd2 alleles of each of the 15 genes in this locus. While transfection with 13 of the genes did not change ISPA-28 affinity, complementation to add the HB3 allele of 2 related genes, *clag3.1* and *clag3.2*, yielded an intermediate phenotype, as expected if both parental channel types are expressed on the host membrane [36]. Allelic exchange and a single nonsynonymous mutation at a highly conserved residue in the leupeptin-resistant PSAC mutant further supported a primary role of the encoded CLAG3 protein. CLAG3 localized to the erythrocyte membrane consistent with the site of PSAC activity. A small variant motif was found to be exposed at the host cell surface and later shown to account for the differential block by ISPA-28 and other clone-specific inhibitors [59,60,61]. Variation at this site strongly suggests selection against the exposed CLAG3 loop by host immune responses [62,63]. Immune selection is also supported by the high levels of anti-CLAG3 antibodies in endemic populations and by the epigenetic silencing of *clag* genes [64,65,66,67,68].

Further evidence for a CLAG3 role in PSAC formation came from independent genetic mapping studies [60,69] and from the epigenetic silencing of CLAG3 and CLAG2, a paralog encoded by a gene on parasite chromosome 2, in a blasticidin S-resistant PSAC mutant [67,70]. These and other CLAG paralogs were known to associate with RhopH2 and RhopH3, two unrelated proteins encoded by single-copy genes conserved in *Plasmodium* spp. [71,72,73]. Several groups have examined these genes and their encoded proteins for possible contributions to PSAC formation. While *clag3*-knockouts can be produced and propagated in nutrient-rich parasite culture media [60], *rhoph2* and *rhoph3* could not be disrupted using CRISPR-Cas9 with several high-scoring sgRNAs that efficiently cleave the genome to allow for gene editing [74]. Conditional knockdowns of these genes were therefore produced, revealing that both RhopH2 and RhopH3 are trafficked to the host membrane and are essential for PSAC formation [74,75,76]. RhopH3, but not CLAG3 or RhopH2, also contributes to host cell invasion. Biochemical studies suggest that each subunit is essential for PSAC formation but that CLAG3 is dispensable because its paralogs—CLAG2, CLAG8 and CLAG9 in *P. falciparum*, with at least two paralogs encoded by each examined *Plasmodium* species—can compensate for the loss of CLAG3. Consistent with this model, RhopH2 and RhopH3 cannot be disrupted as they are encoded by single-copy genes in all *Plasmodium* spp.

These studies have provided compelling evidence for parasite genetic elements and implicated the above gene products, which together form the RhopH complex. These findings are remarkable as workers had previously assumed this complex functioned in either cytoadherence or erythrocyte invasion [77,78,79,80]. They are also surprising because none of the subunits have homology to known channel proteins from higher organisms. Most fundamentally, they also lack the number of predicted transmembrane domains generally thought to be present in channel-forming proteins (Figure 2B). A tantalizing hint comes from the single amphipathic transmembrane domain detected in CLAG3, where a helical wheel analysis reveals that polar residue side chains align at one face of the α-helical transmembrane domain with hydrophobic residues at the face [81]. This arrangement, confirmed by the subsequent determination of the RhopH complex structure by cryo-EM [82], is often found in pore-forming proteins, suggesting the direct formation of the PSAC pore by these unusual proteins. Immunofluorescence studies as well as live-cell FRET reveal that all three members of the RhopH complex traffic together to the host membrane and that at least two of the subunits, RhopH2 and CLAG3, remain tightly associated after insertion at the host membrane [83], further supporting a model in which these unusual parasite proteins form an ion channel. 

At the same time, conclusive evidence for direct PSAC formation by RhopH proteins is still missing. Biochemical studies reveal that the RhopH complex is manufactured as a soluble complex and that it is trafficked and eventually inserted at the host membrane [82]. In contrast to the unrelated parasite RON2 protein [84,85], the RhopH components are not inserted into the host membrane during merozoite invasion. Instead, the soluble complex is deposited into the parasitophorous vacuole at invasion, trafficked across the vacuolar membrane via PTEX [82], and inserted into the host membrane many hours after invasion to form the nutrient uptake channel. Consistent with this, a de novo structure of the soluble complex, determined by cryo-EM microscopy using protein purified from an engineered *P. falciparum* line cultivated in human erythrocytes (Figure 2C, [82]), revealed that the single transmembrane domains of each subunit are buried in the trafficking complex. This finding suggests marked conformational changes associated with insertion into the erythrocyte membrane. These findings were also confirmed without the purification of the RhopH complex using a novel cryo-ID method [86,87]. 

Importantly, the structure of the membrane-embedded RhopH complex remains unknown. This could directly implicate these proteins in PSAC formation, provide insights into how solutes permeate through the channel, and elucidate the mechanisms that underly the channel’s remarkable selectivity properties, as discussed below.

Another important question is whether *Plasmodium* spp. activate unrelated channels on infected erythrocytes. While studies with isolate-specific inhibitors and *rhoph* gene knockdowns suggest that PSAC accounts for the uptake of nearly all solutes, Ca^++^ uptake appears to be mediated by a distinct pathway, as described in Section 4 below. As channels may also be activated under unexplored experimental conditions, it is not possible to exclude the presence of additional parasite-associated channels. Indeed, a recent study proposed cAMP-dependent channels activated by parasite sexual stages [88]. 

### 2.3. Unusual Properties of the Encoded Channel

Paralleling the lack of homology with channel genes from other genera and the unusual structural properties of the RhopH proteins, the encoded channel has several unusual properties that distinguish it from all previously characterized ion channels. First, the single-channel conductance, a measure of how many ions pass through an open pore per unit time, is remarkably small for a broad-selectivity channel that passes bulky organic solutes. Generally, such channels have large pores to allow large solutes to navigate the pores, leading to high flux rates. Somehow, the PSAC maintains a low rate of ion passage despite being able to accommodate large solutes of varying sizes, shapes and charges [89]. This small single-channel conductance necessitated the use of patch-clamp solutions containing molar [Cl^−^] and likely accounts for difficulties with the patch-clamp detection of the channel in some laboratories [46]. Second, and even more remarkably, PSAC stringently excludes small Na^+^ ions despite passing much larger organic cations [89]; this Na^+^ exclusion is critical for intracellular parasite growth because higher Na^+^ permeability would lead to the osmotic lysis of infected cells in the bloodstream [90]. 

### 2.4. Essential Role in Nutrient Uptake and a Druggable Target

Since its discovery, multiple studies have proposed various roles for the increased permeability of infected cells to diverse solutes. These include (1) nutrient acquisition for the developing intracellular parasite [1,3,4,5,91], (2) cation remodeling to raise [Na^+^] and lower [K^+^] in the host cell cytosol [2,92,93], (3) the volume regulation of infected cells by allowing for an efflux of excess amino acid production through hemoglobin digestion [94], (4) the timed osmotic lysis of infected cells to allow daughter parasite egress from infected cells at the end of the intracellular cycle [93], and (5) a nonessential byproduct of infection and intracellular parasite metabolic activity [46]. Each of these proposals had some merit and was based in an understanding of parasite biology, but experimental evidence was missing and difficult to obtain prior to the identification of the channel genes. Gene identification and experimental advances have now clarified the roles, as discussed in this section.

A PSAC role in nutrient acquisition would be consistent with the channel’s permeability to sugars, purines, key vitamins, and the essential amino acid isoleucine, all of which are required for parasite development and not available in adequate quantities within uninfected erythrocytes [1]. Although parasite killing by nonspecific PSAC inhibitors supported this and other proposed essential roles [95,96], uncertainties about the mechanism of killing limited interpretation. Indeed, the selection of a resistant mutant with unaltered PSAC activity and inhibitor affinity confirmed that phlorizdin, a commonly used inhibitor, kills parasites through action on unrelated targets [97]. To address this longstanding uncertainty, a modified medium, termed PSAC growth inhibition medium (PGIM), with lower, more physiological concentrations of three key nutrients acquired via the PSAC was developed [69]. In contrast, the standard medium used in most labs, RPMI 1640 supplemented with a lipid source, has most nutrients present at concentrations > 10-fold higher than levels in plasma from healthy donors. While ISPA-28 has weak activity against parasite growth in standard media, growth inhibition studies using PGIM revealed that Dd2 parasites are killed at an 800-fold lower concentration than HB3, paralleling its clone-specific action against the PSAC in these lines. A linkage analysis using Dd2 × HB3 progeny clones and this difference in growth inhibitory activity mapped the *clag3* locus, establishing that the PSAC block accounts for killing by this uniquely specific inhibitor. Other PSAC inhibitors that do not exhibit differential activity against lab clones are also more effective against in vitro parasite growth in PGIM than in standard RPMI 1640-based media, whereas antimalarials acting on unrelated targets have indistinguishable *IC*_50_ values in these media [69]. Importantly, because these studies required the use of a nutrient-optimized medium, they provided the first experimental evidence for an essential role in nutrient uptake. 

The cation remodeling role for the PSAC is based on the observation that infected cells gradually incur a concomitant increase in [Na^+^] and decrease in [K^+^] with intracellular parasite development because of the nonzero PSAC permeability to these cations [2,90,93,98]. The leaking of these cations at the erythrocyte membrane dissipates the outward and inward gradients for these respective ions, as maintained by the host cell Na^+^/K^+^ ATPase pump [99]. This host cytosol cation remodeling was hypothesized to make the erythrocyte more hospitable for parasite growth, possibly by providing an inward Na^+^ gradient for coupled solute uptake at the intracellular parasite plasma membrane [92]. To explore this role, Pillai et al. designed and used a separate modified medium, 4suc:6KCl, that replaces the Na^+^ salts in the RPMI 1640-based medium with K^+^ salts and sucrose to preserve infected cell osmotic stability. Growth studies revealed that this medium supports unabated parasite growth without a need for adaptation, a remarkable finding in light of the marked changes in composition. Because it abolishes Na^+^ and K^+^ gradients across the erythrocyte membrane, parasite cultivation in 4suc:6KCl prevented PSAC-mediated cation leak and cation remodeling, as confirmed with infected erythrocyte ion content measurements [100]. This study revealed unexpectedly low Na^+^, K^+^, and Cl^−^ requirements for parasite development; it also provided compelling evidence against Na^+^-coupled phosphate uptake at the parasite plasma membrane and excluded an essential role of K^+^ signaling in merozoite activation [101]. 

Alternate roles for the PSAC have also been examined. The volume regulation of infected cells by allowing the PSAC-mediated efflux of excess amino acids generated by hemoglobin digestion remains possible [94]. One prediction of this hypothesis is that potent PSAC inhibitors should lead to the osmotic lysis of infected cells because of blocked amino acid efflux; as this has not been observed [69,102], this hypothesis should be considered with some caution. Another hypothesis, the timed osmotic lysis of infected cells, proposes that gradual Na^+^ and K^+^ leak through the PSAC leads to osmotic swelling and lysis ~44 h after invasion; as this coincides with the duration of *P. falciparum’s* intracellular development, osmotic lysis may facilitate parasite egress at the end of the erythrocytic cycle [93]. This role is excluded by the normal developmental cycle in studies using a 4suc:6KCl medium, in which Na^+^ and K^+^ leak are abolished [100]; it is also inconsistent with studies implicating protein kinases in coordinated parasite egress [103,104]. Finally, proposals that the PSAC is a nonessential byproduct of intracellular parasite development are excluded by *rhoph2* and *rhoph3* knockdown and by an advanced drug discovery and development project [74,75,76,102], both of which establish this target’s essentiality for bloodstream malaria parasites.

### 2.5. Drug Discovery and Development Targeting the PSAC

In addition to discovering isolate-specific inhibitors such as ISPA-28, high-throughput screens have identified multiple novel, potent PSAC inhibitors that sterilize in vitro parasite cultures. Iterations of medicinal chemistry and PSAC inhibition measurements with a robust transmittance-based assay have yielded improved target affinity with desirable pharmacokinetic properties for the development of oral antimalarial drugs targeting this unexploited target [102]. The PSAC target has several desirable properties for therapy development. Unusual biochemical and molecular properties distinguish it from mammalian channels, permitting the identification of specific inhibitors without activity against a battery of human channels and transporters and reducing the risk of drug side effects. The PSAC’s surface location on infected cells is also a significant advantage for this target as it ensures target access by drugs in plasma; it also significantly reduces the risk of acquired resistance via drug efflux, a mechanism that has compromised effectiveness for several intracellular parasite targets [105,106]. Gene identification, DNA transfection studies, and cryo-EM structure determination should all help guide the medicinal chemistry optimization of PSAC inhibitors to produce potent and specific derivatives that advance to clinical trials.

## 3. The PVM Channel and PTEX Translocon

### 3.1. Background

**Figure 3:**
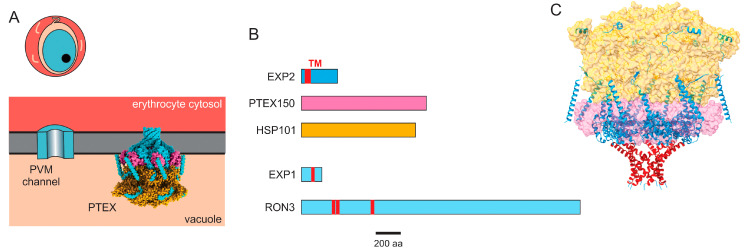
The PVM channel and PTEX. (**A**). Schematic showing the PVM channel and the PTEX translocon at the PVM. (**B**). Ribbon diagrams of core PTEX components, EXP1, and RON3, drawn to scale. Predicted transmembrane domains indicated in red. (**C**). Cryo-EM structure of the PTEX translocon, with core components color-coded as in panel (**B**). The transmembrane domains (red) contributed by each of seven EXP2 monomers form a pore. PDB: 6E11.

A second example of a unique parasite ion and solute channel localizes to the parasitophorous vacuole membrane (PVM, Figure 3A). The PVM is initially formed as an invagination of the erythrocyte membrane during invasion by the merozoite; it grows with the maturing pathogen through the addition of lipids and proteins [107,108,109]. As it is an intracellular membrane for which there are no robust methods for isolation or purification, macroscopic transport methods such as tracer accumulation cannot be reliably used to characterize PVM transport properties. Patch-clamp methods, though complicated by the small size of the intracellular parasite, are therefore better suited for the study of PVM transport. Indeed, they provided the first direct measurements of PVM ion transport as they identified a single-ion channel type having a large conductance [110]. This PVM channel is present at a high copy number and is primarily open at the resting membrane potential. It is also nonselective to ions and solutes of any charge and size up to 1400 dal in size based on pore exclusion studies with polyethylene glycols [111]. Remarkably, fluorescent dye exclusion studies of fibroblasts infected with *Toxoplasma gondii*, a distantly related parasite that causes toxoplasmosis in humans and animals, revealed pores on that parasite’s PVM with a nearly identical size [112]; similar dye exclusion studies suggest that *Eimeria nieschulzi*, a distantly related parasite that produces intestinal disease in brown rats, also has such pores [113]. These observations indicate that the PVM is a molecular sieve for small solutes, a feature that appears to be highly conserved in divergent protozoan parasites with intracellular development.

The *P. falciparum* PVM must also mediate the export of parasite proteins into the host cytosol. These larger macromolecules serve various effector functions in the host cytosol, with some integrating into Maurer’s cleft and host erythrocyte membranes [36,114,115,116]. Initially, the study of this protein export proceeded independently of the above PVM ion and solute flux studies. Our understanding of this process evolved from initial computational studies, revealing that most exported proteins carry a recessed PEXEL motif (RxLxE/D/Q) downstream of the ER signal sequence [117,118]. A pioneering study then used a GFP reporter protein fused to the mouse dihydrofolate reductase (mDHFR) protein to visualize protein export at the PVM and found that this chimeric reporter protein could not be exported upon the addition of high-affinity folate analogs that prevent mDHFR unfolding [119]. These findings implicated a protein-conducting pore and revealed that proteins must be unfolded, presumably by one or more chaperones, to cross the PVM. This finding is also consistent with early studies showing that protein export is an ATP-dependent process [120].

### 3.2. Identification of PTEX Components and Other Proteins Involved in Export

The molecular determinants of the protein export translocon were uncovered through a proteomic approach that capitalized on the above findings. Recognizing that an ATP-dependent chaperone is likely involved, de Koning-Ward et al. examined the detergent-resistant membrane proteome of immature infected cells to identify HSP101, a ClpA/B-like AAA + ATPase chaperone protein [121]. Immunoprecipitation experiments were then used to identify associated proteins, PTEX150, EXP2, PTEX88, and TRX2. The interactions between these proteins were confirmed through reciprocal pull-down experiments. Importantly, this complex, termed the *Plasmodium* Translocon of EXported proteins (PTEX), was then shown to interact with exported proteins containing the PEXEL motif through mass spectrometry studies, providing compelling evidence for its role as a translocon. Based on biochemical studies showing that EXP2 is integral to membranes and that it has structural homology to pore-forming hemolysin E, EXP2 was proposed to be the pore-forming subunit of the putative translocon in this foundational study [121]. 

A direct functional link between the PTEX protein complex and the export of effector parasite proteins then came through conditional knockdown studies of HSP101 and PTEX150. In one study [122], the conditional knockdown of the *P. falciparum* HSP101 prevented the export of various PEXEL-containing proteins into the erythrocyte cytosol; several proteins that lack PEXEL domains but are known to be exported (termed PEXEL-negative exported proteins or PNEPs [123]) were also blocked from export, indicating that they also require an intact and functional PTEX translocon. Interestingly, CLAG3 export was found to be unaffected in this study, but PSAC activity was abolished; a later study using the same antibodies and parasite line contradicted this, finding that the export of CLAG3 and other RhopH proteins is abolished by PTEX knockdown, paralleling the failure to induce PSAC activity [74]. In a second study [124], HSP101 knockdown in the *P. berghei* rodent malaria parasite confirmed the blocking of protein export and established the translocon’s importance under in vivo conditions. These workers also performed PTEX150 knockdown in *P. falciparum*, revealing that this subunit is also essential for translocon activity. Together, these two studies revealed a failure of intracellular parasite maturation, inhibited in vitro and in vivo parasite growth, and the blocking of progression into gametocyte stages as required for transmission via mosquitoes. 

In contrast to EXP2, PTEX150, and HSP101, the two other PTEX subunits identified by pull-down studies, TRX2 and PTEX88, can be genetically deleted. These knockout parasites exhibit slowed growth along with compromised sequestration and virulence in *P. berghei*-infected mice [125,126,127,128]. These findings may reflect the accessory roles of these two components as some experiments reveal a reduction in the export of proteins involved in infected cell cytoadherence [124,128]. Nevertheless, whether TRX2 and PTEX88 contribute to protein export or serve unrelated roles in intracellular parasite development remains to be conclusively elucidated. 

Two proteins not detected in the initial PTEX component pull-down studies also appear to contribute to protein and solute transport at the PVM. The first of these, RON3, was discovered when its conditional knockout exhibited failed intracellular parasite maturation and blocked protein export at the PVM [129]. This study also used 2-NBDG, a labeled glucose analogue, to obtain indirect evidence linking RON3 to the PVM ion and nutrient uptake channel (Figure 3B). A subsequent study confirmed these findings and also implicated a RON3 role in erythrocyte invasion [130]; this study also reported compromised PSAC activity upon RON3 knockdown, presumably due to the reduced export of RhopH proteins.

The second protein, EXP1, was also implicated through conditional knockout studies [131] (Figure 3B). In contrast to PTEX core components and RON3, EXP1 ablation did not compromise the export of parasite proteins into the host cytosol. At the same time, patch-clamp studies revealed a near-complete loss of PVM channel activity; a reduced tolerance to amino acid deprivation suggested that this channel mediates nutrient uptake into the parasitophorous vacuole for parasite utilization. Subsequent studies revealed that EXP1 knockdown compromises the PVM ultrastructure and EXP2 distribution on the PVM [132]; it also appears to reduce nutrient and drug uptake at the PVM in indirect transport measurements [133]. 

### 3.3. PTEX Translocon Structure

The de novo cryo-EM structure of the PTEX translocon, solved using protein complexes purified from blood cultures [134], then revealed a pore formed by seven EXP2 monomers. Immediately above the pore’s funnel, seven PTEX150 protomers were apparent, with a hexameric HSP101 protein-unfolding motor at the top of the complex (Figure 3C). The accessory PTEX88 and TRX2 proteins were not visualized in the structure, adding to uncertainties about these proteins’ roles. Importantly, the complex was captured with unfolded cargo protein in transit through the pore. Two discrete conformations of this cargo-associated complex, designated as “engaged” and “resetting”, strongly implicate the energy-driven threading of cargo through the PVM. These findings provide a structural mechanism for protein export and open the door to structure-guided therapy development. 

### 3.4. Transport Studies Linking the Translocon to the PVM Channel

An important question has been whether the molecular basis of the PVM ion channel discovered in patch-clamp studies is the same as that of the PTEX translocon, which has been studied primarily using protein imaging. To address this question, Garten et al. used engineered parasites carrying either EXP2 conditional knockdown or overexpression and performed cell-attached patch-clamp experiments [135]. These experiments lines revealed a clear correlation between EXP2 expression and PVM ion channel abundance, using methods similar to those used previously [74,110]. Patch-clamp experiments with parasites carrying a C-terminal EXP2 truncation mutant revealed modest but statistically significant changes in the PVM channel’s voltage dependence, suggesting a direct link between EXP2 and the functional PVM channel. Based on immunoprecipitation and stage-specific expression studies, this study proposed that EXP2 exists in two forms on the PVM: the PTEX protein translocon that maintains stable associations with PTEX150 and HSP101 and a distinct heptameric pore not associated with other PTEX components that functions as the PVM ion and nutrient channel [135].

Support for this interesting model comes from studies of the PVM channel ortholog in *Toxoplasma gondii* [112]. A computational analysis revealed two proteins, TgGRA17 and TgGRA23, with sequence homology to EXP2 [136]. The knockout of TgGRA17, but not TgGRA23, produced *T. gondii* parasites with abnormal PVM morphology in infected fibroblasts; this phenotype was rescued by complementation with *P. falciparum* EXP2, supporting functional orthology. In contrast to EXP2 knockdown, the disruption of TgGRA17 or TgGRA23 did not compromise protein export by *T. gondii*. Dye uptake studies in infected fibroblasts and the patch clamping of *Xenopus* oocytes expressing TgGRA17 or TgGRA23 also supported a role for these proteins in the formation of the Toxoplasma PVM channel, though these data were limited by the incomplete effects of complementation and modest patch-clamp currents [136]. Also in partial support of this model, a recent study found that TgGRA17 expression in *P. falciparum* could not adequately complement EXP2 knockdown despite the use of appropriate promoters and chimeric protein constructs that could allow for associations with other PTEX components [137].

Although these studies suggest that EXP2 forms the PVM channel, several uncertainties remain. In addition to the issues raised above, we can ask what functions RON3 and EXP1 serve in the formation of the PVM channel given their transmembrane domain topologies (Figure 3B), localization to the PVM, and transport studies suggesting involvement in ion and solute flux [129,131]. In light of technical challenges associated with PVM patch clamping [44], the limitations of DNA transfection studies, and the complex interactions between proteins at the PVM, new technologies may be required to conclusively define the roles played by these various proteins.

Regardless of their precise molecular determinants, the PVM channel and the PTEX protein export machinery are both exciting targets for antimalarial therapy development. Although direct inhibitors of these activities are presently unavailable, a potent chemical mimetic of the PEXEL motif that targets proteins for export, WEHI-842, effectively kills bloodstream parasites by preventing processing required for export [138], providing proof of concept for transport inhibition at this membrane as an important future direction for drug discovery and development.

## 4. Other Channel Activities That May Be Encoded by Novel Parasite-Specific Genes

In addition to the above relatively well-characterized parasite channels, there are additional *P. falciparum* transport activities suggestive of pathogen-specific channels. Increased Ca^++^ permeability at the host erythrocyte membrane is an important example identified through tracer flux [8,139,140]. ^45^Ca^++^ uptake measurements reveal that greater uptake by infected cells cannot be explained by either the downregulation of the Ca^++^ extrusion pump or the stimulation of the passive Ca^++^ carrier endogenous to erythrocyte membranes [141,142,143]. While the PSAC is responsible for the increased permeability of other ions and solutes after infection, it does not account for increased Ca^++^ permeability as specific PSAC inhibitors do not reduce Ca^++^ transport at the infected erythrocyte membrane [9,10]. These observations suggest a distinct parasite-induced Ca^++^ transporter. Based on kinetic studies and rare events detected in patch-clamp experiments, this transporter appears to be an ion channel [141]. It is not blocked by the known blockers of mammalian Ca^++^ channels, but its activation after infection is compromised by PTEX knockdown [10]. Taken together, these findings suggest a parasite-derived channel that serves an essential role in Ca^++^ uptake and utilization by the intracellular pathogen [7].

Novel parasite ion channels are also likely present at other membrane barriers encountered throughout the parasite life cycle (Figure 1). Intracellular membranes, such as that of the parasite digestive vacuole or the multiple membranes surrounding the apicoplast [144,145], must have channels to mediate solute exchange and sustain organellar biochemical activities. Parasite stages that are not intracellular, such as bloodstream merozoites and ookinetes in the mosquito midgut, also require rapid exchanges with their extracellular environment for signaling, motility, nutrient uptake, and metabolic waste removal. 

The paucity of ion channel gene orthologs identified through the whole-genome sequencing of *Plasmodium* spp. is indeed surprising. Transport studies and directed gene identification strategies, such as those successfully used for the PSAC and PTEX, should provide fundamental insights into parasite biology and uncover important targets for much-needed antimalarial therapies. 

## Data Availability

No new data were created or analyzed in this study. Data sharing is not applicable to this article.
